# Modeling of gonioscopic anterior chamber angle grades based on anterior segment optical coherence tomography

**DOI:** 10.1186/s40662-020-00196-1

**Published:** 2020-06-02

**Authors:** Yingying Dai, Shaodan Zhang, Meixiao Shen, Yuheng Zhou, Mengyi Wang, Jie Ye, Dexi Zhu

**Affiliations:** School of Ophthalmology and Optometry, Wenzhou Medical University, Wenzhou, Zhejiang China

**Keywords:** Anterior chamber, Imaging, Glaucoma

## Abstract

**Background:**

To quantitatively assess anterior chamber angle (ACA) structure by anterior segment optical coherence tomography (AS-OCT) and develop a model to evaluate angle width as defined by gonioscopy.

**Methods:**

The ACAs of each quadrant were evaluated by gonioscopy, classified by the Scheie grading system, and assigned into one of the three grades: small angle (SA), moderate angle (MA), and large angle (LA). The eyes were imaged by AS-OCT, and ACA structural parameters including angle opening distance at the scleral spur (AODSS) and at 750 μm anterior to the scleral spur (AOD750), trabecular-iris space area at 750 μm anterior to the scleral spur (TISA750), and a newly defined parameter “light intersection distance” (LID), were measured. The ACA structural data were used to construct an ordered logistic regression model for assignment of ACAs to one of the three angle grades. The validity of the model was then tested.

**Results:**

A total of 169 quadrants from 53 subjects were included in the analysis, of which 111 quadrants were included in the modeling data and 58 in the testing data. In pairwise comparisons of SA, MA, and LA by ANOVA, the measured parameters were as follows: AOD750 (0.174 ± 0.060 vs. 0.249 ± 0.068 vs. 0.376 ± 0.114 mm; *P* < 0.001), TISA750 (0.075 ± 0.035 vs. 0.117 ± 0.036 vs. 0.181 ± 0.062 mm^2^; *P* < 0.001), and LID (− 0.300 ± 0.187 vs. -0.085 ± 0.170 vs. 0.122 ± 0.156 mm; *P* < 0.001). The ACA grading model based on LID showed a relatively high correction rate of 72.4%, and the model efficiency, calculated using the receiver operating characteristic, showed an area under the curve of 0.740. Weighted kappa statistics showed a good agreement for multiple ACA grades (0.772).

**Conclusions:**

The AS-OCT-based multiple ACA grades model was demonstrated as a non-contact approach for ACA assessment with high speed and high spatial resolution, providing guidance for diagnosis of angle closure.

## Background

Primary angle closure glaucoma (PACG), characterized by anterior chamber angle (ACA) closure, increased intraocular pressure (IOP) and glaucomatous optic neuropathy, is the leading cause of irreversible blindness in Asia, which imposes a significant burden on health care systems and societies [1–5]. Of the numerous subtypes of glaucoma, PACG has higher visual morbidity [6], therefore early prophylactic treatment may be indicated in patients at risk of vision loss due to occludable angles [7], though a recent large-sample study showed that the incidence of primary angle closure was relatively low among patients of suspect angle closure [8]. Currently, gonioscopy, as the gold reference standard, is the most commonly used clinical examination to evaluate the ACA for the purpose of both diagnosis and early intervention [9, 10]. The anatomical structure of the ACA is observed directly by gonioscopy, and the deepest visible structure of the ACA is commonly recorded in the form of structure name or degree in the four quadrants, separately. However, the examination requires contact with the cornea, and the unquantified outcome may be affected by the cooperation of patients and subjective judgment of clinicians [11, 12]. A recent study reported a good agreement for gonioscopy when distinguishing opening angles from closed ones, but a fair to moderate agreement when assessing the specific angle structures among the experienced optometrist [13]. Thus, an alternative examination is necessary for efficient and effective assessment of the ACA.

**Fig. 1:**
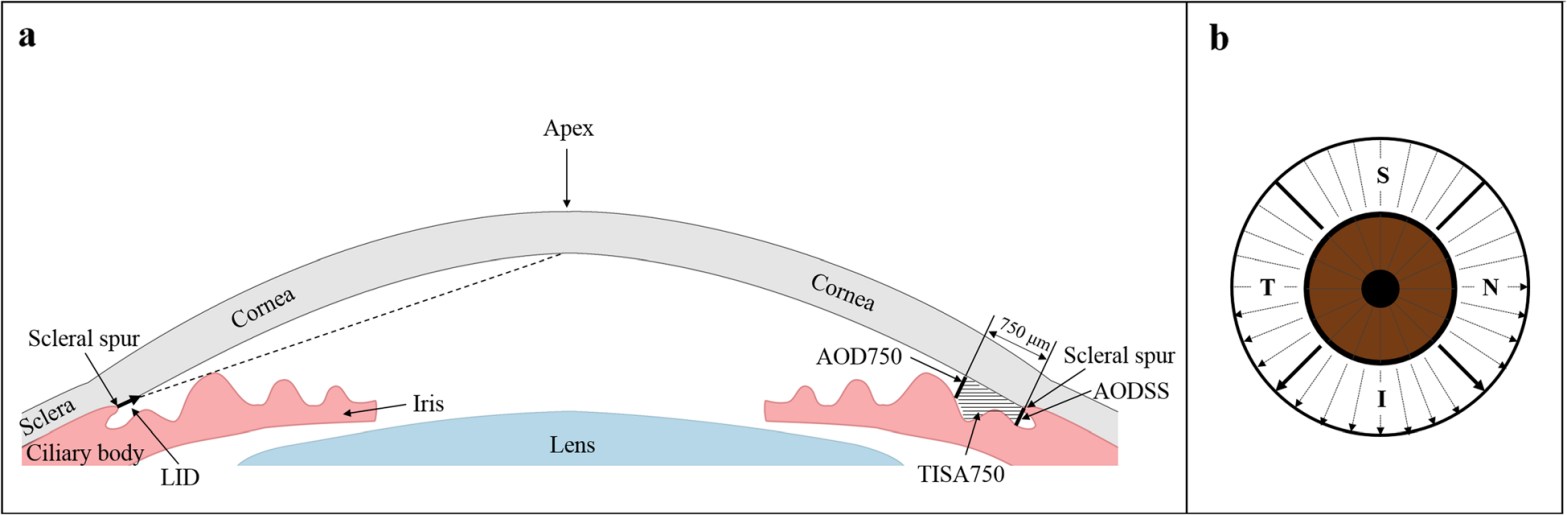
Measurement of anterior chamber angle parameters with AS-OCT. **a** Illustration of parameters used in the study. **b** Scanning mode and quadrant distribution of the right eye. LID, light intersection distance; AODSS, angle opening distance at the scleral spur; AOD750, angle opening distance at 750 μm; TISA750, trabecular-iris space area at 750 μm; S, superior quadrant; I, inferior quadrant; T, temporal quadrant; N, nasal quadrant

Anterior segment optical coherence tomography (AS-OCT) provides a non-contact and quantifiable method of evaluating the ACA, allowing deeper penetration and high-resolution imaging of the structure [14, 15]. Previous studies have revealed the relationship between AS-OCT–based parameters and angle closure. Two types of AS-OCT–based parameters were identified as global parameters and peripheral parameters. The global parameters characterize the morphology of anterior segment as a whole, including anterior chamber width [16], anterior chamber area [17], volume [17], and lens vault [18]. The peripheral parameters characterize the morphology on the ACA especially, including the angle opening distance (AOD) and the trabecular-iris space area (TISA) [19, 20]. The AOD is defined as the perpendicular distance between the trabecular meshwork and the iris at 750 μm anterior to scleral spur, which is designated as “AOD750” (Fig. 1a) in this study. The TISA is defined by four boundaries, i.e., anteriorly, the AOD750; posteriorly, a perpendicular line drawn from the scleral spur to the iris; superiorly, the inner corneoscleral wall; and inferiorly, the iris surface. In this study, it is designated as “TISA750” (Fig. 1a). Both the global and the peripheral parameters have been confirmed to contribute to angle closure [21, 22]. Compared with the global parameters, the peripheral ones reflect a more similar observation as the gonioscopic findings of each quadrant. However, there was no strong evidence to explain the relationship between the AS-OCT findings and the multiple grades of the ACA by gonioscopy, for instance, grades I – IV of the Scheie grading system [23–25] (grade I, visible ciliary body; grade II, visible scleral spur; grade III, visible anterior trabecular meshwork; grade IV, angle structures not visible). This could be due to the emphasis on the differentiation of open from closed ACA and the absence of definitive correlations between the known angle parameters and the different grades of chamber angles in previous studies [26, 27].

The purpose of this study was to find an efficient parameter to detect the grade of the ACA and establish an AS-OCT-based multiple ACA grades model that associates 360 degrees of ACA, based on gonioscopic grouping. The model should indicate the distribution of ACAs in the eye and provide guidance for clinical diagnosis.

## Methods

### Subjects

Subjects (≥ 40 years old) were recruited consecutively from the glaucoma clinic of Wenzhou Eye Hospital, including both narrow-angle and open-angle patients. Written informed consent was obtained from all subjects. The work was carried out according to the World Medical Association’s Declaration of Helsinki and approved by the Ethics Committee of Wenzhou Medical University (2019-027-K-26). Suspected or confirmed primary angle closure and PACG were included as narrow angles (defined as the invisibility of the posterior pigmented trabecular meshwork at least 180° on static gonioscopy) [7], while subjects with a diagnosis of primary open angle glaucoma, ocular hypertension, and cataracts were included as open angles. Subjects with a history of intraocular surgery or any corneal diseases, such as a corneal nebula that would block AS-OCT imaging were excluded from the study. However, patients who had been treated with laser peripheral iridotomy were included. The right eyes were recruited if both eyes of a single subject were eligible. After providing their medical history, all subjects underwent the following examinations on the same day: measurement of visual acuity, non-contact tonometry, slit-lamp biomicroscopy, gonioscopy, and anterior segment imaging by AS-OCT. Both gonioscopy and AS-OCT were performed in a dark room.

### Gonioscopy and grouping

In all cases, gonioscopy was performed by a single experienced ophthalmologist using a Goldmann 1-mirror lens (Ocular Instruments., Bellevue, WA, USA) at high magnification (× 16) with eyes in the primary gaze position. Indentation gonioscopy was performed using a Sussman 4-mirror lens (Ocular Instruments Inc., Bellevue, WA, USA). A 1-mm light beam was reduced to a very narrow slit and offset horizontally to assess the superior and inferior quadrants, and vertically for the nasal and temporal quadrants. Care was taken to avoid accidental indentation and to avoid light falling on the pupil during the examination. The grade of ACA in each quadrant was classified and recorded with the Scheie grading system [23–25], which was commonly used in the glaucoma clinic of Wenzhou Eye Hospital. According to the diagnosis and the risk of angle closure, quadrants with open angles and grades I were assigned to the large angle (LA) group, while quadrants of grades II were assigned to the moderate angle (MA) group, and quadrants of grades III and IV were assigned to the small angle (SA) group.

### AS-OCT

A custom-built swept-source OCT was employed to acquire the anterior chamber images. This system used the vertical cavity surface-emitting laser (VCSEL) light source (Thorlabs SL130V1–20,024, Newton, NJ, USA) with a center wavelength of 1300 nm and a scan rate of 200 kHz. The axial resolution was 5.7 μm, and the radial scan range and axial scan range were of 17 mm and 5.86 mm in tissue, respectively. Subjects were asked to focus on the internal fixation image in the primary gaze position. Using the radial scanning mode (Fig. 1b), 16 radial B-scans with 11.25° intervals were acquired over 0.9 s. Both upper and lower eyelids were softly pulled apart, avoiding inadvertent pressure on the globe, to ensure that the whole anterior segment was photographed by the device.

The operator who performed the AS-OCT was masked to the clinical data. The same operator, using our custom-made program [28], then processed the images. The locations of the scleral spur at both sides in each image were determined manually by the same operator based on the description of it as an inward protrusion of the sclera with a change in curvature of the inner surface [29]. Even though the scleral spur may not always be visible in each scanning image, the position of the scleral spur can be traced with the adjacent images with clear visibility of it. Boundaries, including the anterior and posterior surfaces of the cornea and the anterior surface of iris and lens, were automatically optically corrected and segmented based on the dynamic programming. The anterior segment parameters, including the AOD750, TISA750, angle opening distance at the scleral spur (AODSS), and the light intersection distance (LID) were then measured automatically from the boundary data (Fig. 1a). AODSS was defined as the perpendicular distance between the iris and the plane of the inner scleral wall from the scleral spur. LID, based on the optical path of the gonioscopic lens, was defined as the vector from the scleral spur to the specific point on the posterior corneal surface in the primary gaze position, which is derived from the intersection point of the line passing from the corneal endothelial apex that is tangential to the vertex of the iris. The value of LID was defined as negative if the intersection point was located anterior to the scleral spur, and positive if the intersection was posterior to the scleral spur.

### Statistical analysis

Eight scanning intervals were divided in each quadrant according to the scanning mode of the AS-OCT instrument (Fig. 1b). The mean values of each quadrant were derived from the data over the average for the whole quadrant, and each quadrant was treated as an independent sample as a with-in variable which was assessed by means of the generalized estimating equation. Bland-Altman plots was used to assess both intraobserver and interobserver agreement. Intraobserver agreement of the ACA parameters were calculated with 18 quadrants by the experienced operator in two operations with a 2-min interval using AS-OCT, while interobserver agreement of ACA parameters were evaluated by two operators (the experienced operator and nonexpert) independently. Categorical data was compared using chi-squared tests, while ANOVA was used for continuous data. The area under the curve (AUC) of the receiver operating characteristic (ROC) was used to calculate the diagnostic performance of every parameter in two-by-two comparisons.

To establish the AS-OCT-based classification model, we applied a stepwise regression analysis that was followed by an ordered logistic regression analysis to obtain the model. The logistic regression value of the probability were expressed as follows:

$$ {Q}_{SA}=\log\ it\left({P}_{SA}\right)={\alpha}_1-{\beta}_1\ast {X}_1-{\beta}_2\ast {X}_2-\dots -{\beta}_n\ast {X}_n $$

$$ {Q}_{SA+ MA}=\log\ it\left({P}_{SA}+{P}_{MA}\right)={\alpha}_2-{\beta}_1\ast {X}_1-{\beta}_2\ast {X}_2-\cdots -{\beta}_n\ast {X}_n $$

where *Q*_*SA*_ was the logistic regression value of the probability calculation for SA, while *Q*_*SA + MA*_ was the value of the cumulative probability calculation for both SA and MA. *P*_*SA*_ and *P*_*MA*_ were the probabilities of a measured angle belonging to the SA and MA group, respectively, *α*_*1*_ and *α*_*2*_ were constants generated by SPSS (version 22, SPSS Inc., Chicago, IL, USA), *β* was the regression coefficient, *X* was the value of the angle parameter, n was the number of the angle parameter involved. Then, the respective probabilities of three groups for each quadrant were calculated:

$$ {P}_{SA}=\mathit{\exp}\left({Q}_{SA}\right)/\left(1+\mathit{\exp}\left({Q}_{SA}\right)\right) $$

$$ {P}_{MA}=\exp \left({Q}_{SA+ MA}\right)/\left(1+\exp \left({Q}_{SA+ MA}\right)\right)-{P}_{SA} $$

$$ {P}_{LA}=1-\left({P}_{SA}+{P}_{MA}\right) $$

where *P*_*LA*_ was the probability of LA.

Three probabilities for three ACA groups were obtained by measured parameters. Then each quadrant was assigned to the corresponding group with the highest probability among *P*_*SA,*_*P*_*MA*_ and *P*_*LA*_. The correct rate of the data was determined by the number of quadrants classified in the correct group. To calculate the model performance, ROC was used based on the validation data set including 58 quadrants selected randomly. Grouping results of gonioscopy and the multi-grade model were compared using the weighted Fleiss’s kappa. *P*<0.05 was considered as statistically significant.

## Results

**Fig. 2:**
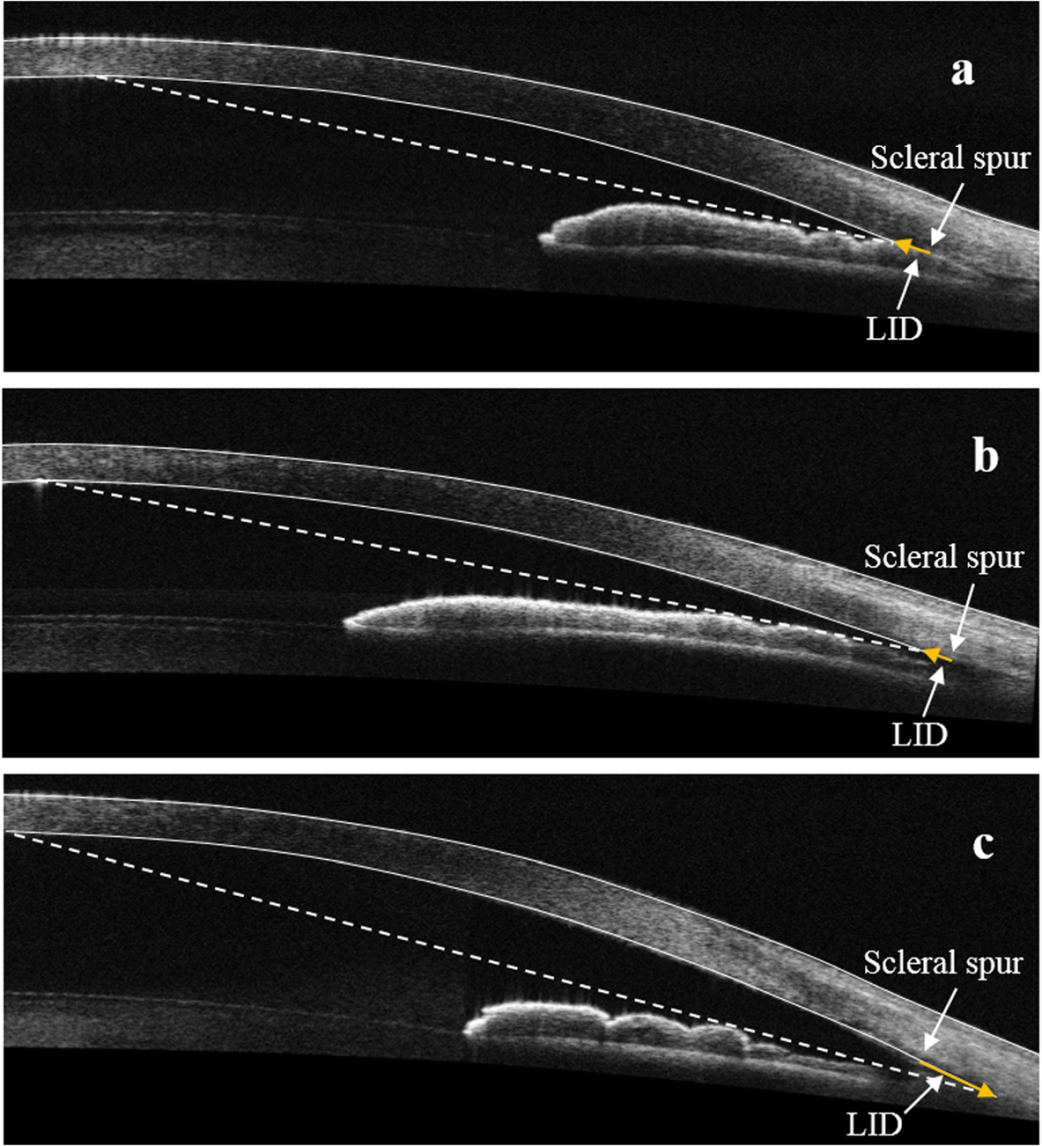
Typical AS-OCT images of 3 quadrants and the corresponding LID. **a** small angle; **b** moderate angle; **c** large angle. LID, light intersection distance

A total of 212 quadrants from 53 patients were recruited in this study, of which 17 quadrants were excluded due to incomplete imaging and 26 were excluded for poor identification of scleral spur. Therefore, data from 169 quadrants were available in the analysis. There were 18 primary angle closure suspects, 6 primary angle closure (5 had laser peripheral iridotomy), 8 PACG, 3 primary open angle glaucoma, 2 ocular hypertension, 12 cataracts and 4 healthy subjects. All ACA parameters of each group were Gaussian distributed. The generalized estimated equation was used to assess the contribution of quadrants from a single eye as quadrants from the same eye might be correlated. The results showed that no significant inter-quadrant difference was found for these ACA parameters (P_AODSS_ = 0.589, P_AOD750_ = 0.778, P_TISA750_ = 0.820, P_LID_ = 0.606), suggesting that there was no correlation between different quadrants in one eye if assigned in the same group, and therefore each quadrant could be treated as an independent sample. All quadrants were divided randomly into modeling data (111 quadrants) set and testing data set (58 quadrants). The basic information and number of quadrants of each group in modeling data are shown in Table 1 and there was no significant difference between age, gender and IOP of three groups. Typical AS-OCT images obtained during the imaging of each group are shown in Fig. 2 and LID is marked on it. There was no significant difference of inter-quadrant for all anterior chamber parameters used in this study (*P* > 0.05), using generalized estimating equation. ANOVA was performed to calculate angle parameters.

**Table 1:** Comparisons and summaries of the modeling data (*n* = 111)

Variables	SA	MA	LA	*P* Value
Age, years	55.9 ± 8.2	59.9 ± 8.2	59.3 ± 8.1	0.099
Male gender, %	50.0	30.0	47.9	0.204
IOP, mmHg	13.5 ± 5.7	13.8 ± 4.3	14.4 ± 5.2	0.712
Quadrant, N (%)	34	29	48	
Superior	14 (41.2)	5 (17.3)	5 (10.4)	
Inferior	9 (26.5)	7 (24.1)	12 (25.0)	
Nasal	6 (17.6)	7 (24.1)	14 (29.2)	
Temporal	5 (14.7)	10 (34.5)	17 (35.4)	

*SA* = small angle; *MA* = moderate angle; *LA* = large angle; *IOP* = intraocular pressure; *N* = number of quadrants reported

### Intraobserver and interobserver agreement of ACA parameters

**Fig. 3:**
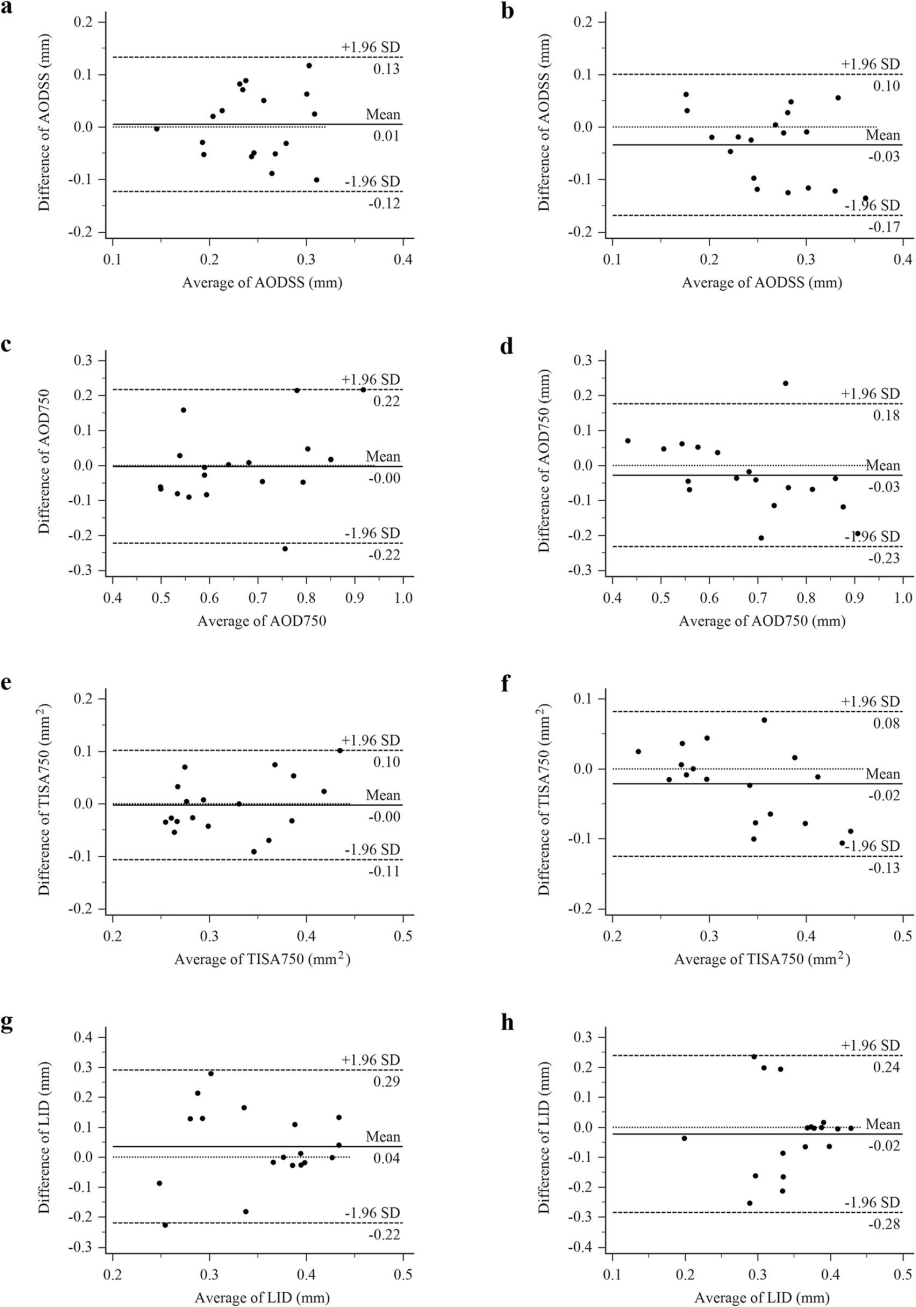
Bland–Altman plots of the agreement of measurements of ACA parameters. **a** intraobserver agreement of AODSS; **b** interobserver agreement of AODSS; **c** intraobserver agreement of AOD750; **d** interobserver agreement of AOD750; **e** intraobserver agreement of TISA750; **f** interobserver agreement of TISA750; **g** intraobserver agreement of LID; h) interobserver agreement of LID; SD = standard deviation

Difference between two measurements of AODSS (Fig. 3a), AOD750 (Fig. 3c), TISA750 (Fig. 3e) and LID (Fig. 3g) were calculated by the experienced operator. The mean ± SD difference with 95% of limits of agreement in identification of these parameters were as follows: AODSS (0.01 ± 0.13) mm, AOD750 (0.00 ± 0.22) mm, TISA750 (0.00 ± 0.10) mm^2^, LID (0.04 ± 0.22) mm. Difference between two operators (the experienced operator and nonexpert) were also calculated. The mean ± SD difference of AODSS (Fig. 3b), AOD750 (Fig. 3d), TISA750 (Fig. 3f), LID (Fig. 3h) were − 0.03 ± 0.1 mm, − 0.03 ± 0.18 mm, − 0.02 ± 0.08 mm^2^ and − 0.02 ± 0.24 mm, respectively. Except AOD750 (94.4%) in both measurements and LID (94.4%) in intraobserver measurement, all the remaining data were in the range of 95% of limits of agreement, demonstrating good agreement of the measurements.

### Comparison of the three ACA groups

**Fig. 4:**
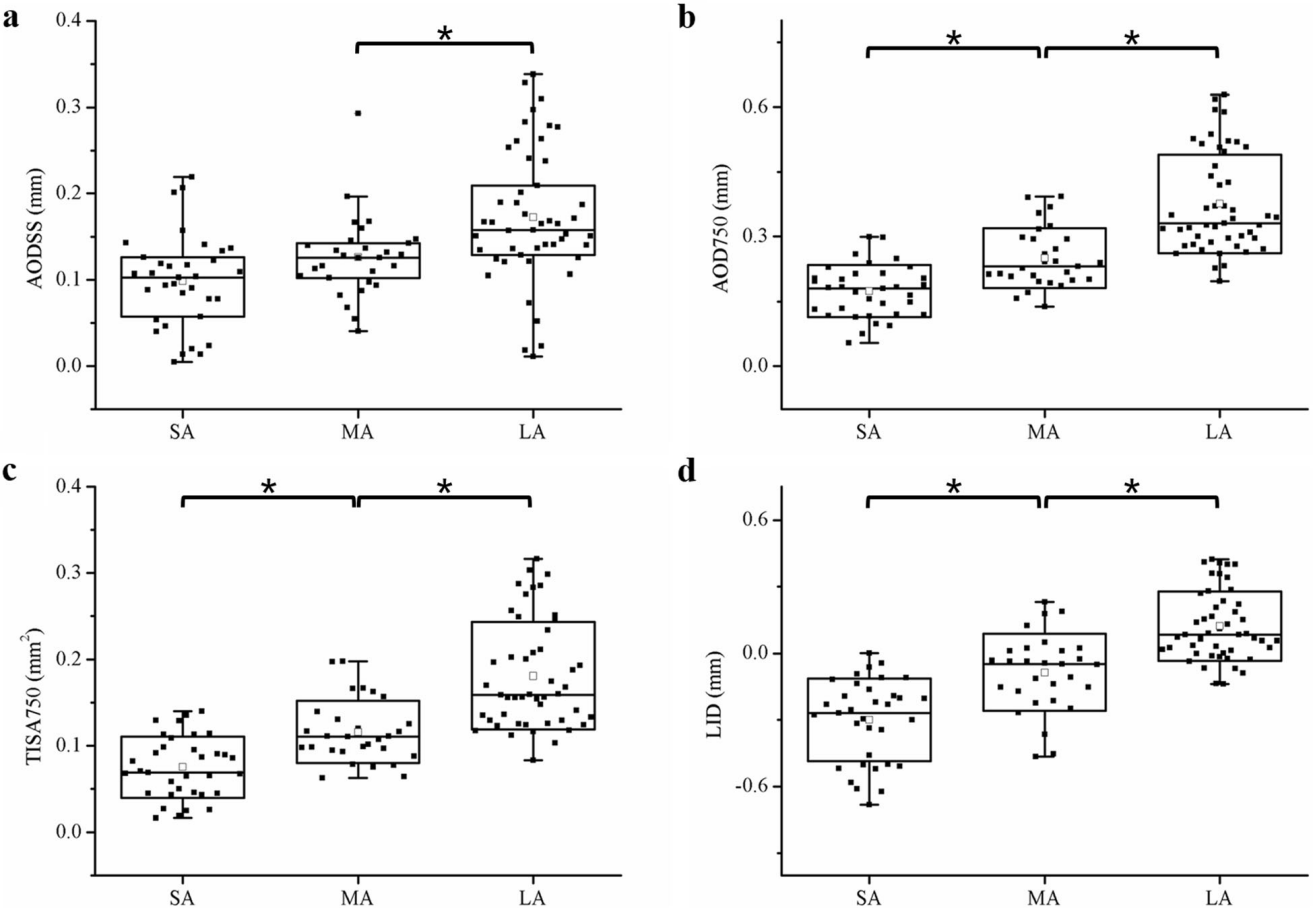
Box plot of comparisons of AODSS (**a**), AOD750 (**b**), TISA750 (**c**), and LID (**d**) between small, moderate, and large ACAs. Data of four parameters are shown in black solid square, the means are shown in black hollow square, and the range of 95% of limits of agreement is shown as box plots. SA, small angle; MA, moderate angle; LA, large angle. AODSS, angle opening distance at the scleral spur; AOD750, angle opening distance at 750 μm; TISA750, trabecular-iris space area at 750 μm; LID, light intersection distance; *, *P* < 0.001

Generally, AOD750, TISA750, and LID were smaller in narrower ACAs than in larger ACAs (*P* < 0.001, Fig. 4). While AODSS in the SA group was not significantly smaller than the MA group (*P* = 0.203), it was smaller in the MA group than in the LA group (*P* < 0.001). ROC analysis was performed to compare the diagnostic efficiency of the different parameters (Table 2). The AUCs for AOD750, TISA750, and LID were larger than the AUC for AODSS in group pairwise comparisons.

**Table 2:** AUC of different parameters in pairwise comparisons

	AODSS	AOD750	TISA750	LID
SA vs. MA	0.662	0.794	0.782	0.811
MA vs. LA	0.729	0.835	0.833	0.824

*AUC* = area under the curve; *SA* = small angle; *MA* = moderate angle; *LA* = large angle; *AODSS* = angle opening distance at the scleral spur; *AOD750* = angle opening distance at 750 μm; *TISA750* = trabecular-iris space area at 750 μm; *LID* = light intersection distance

### Establishment of the AS-OCT-based multiple ACA grades model

To generate the AS-OCT-based multiple ACA grades model, AODSS (*P* = 0.222), AOD750 (*P* = 0.267) and TISA750 (*P* = 0.163) were excluded during the stepwise regression analysis, while LID was selected to establish the model (*P* < 0.001). The classification model was shown in the following equations:

$$ {Q}_{SA}=\log it\left({P}_{SA}\right)=-2.111-10.231\ast LID $$

$$ {Q}_{SA+ MA}=\log it\left({P}_{SA}+{P}_{MA}\right)=0.019-10.231\ast LID $$

A separate cohort of 58 quadrants was acquired to validate the precision of the model (Table 3). There was no significant difference in age or gender of the three grade groups. Using the LID values, the number of the quadrants in the three ACA groups were calculated with formulas developed in the modeling data (Table 4). The total correct rate of the testing data was 72.4%, and the rates were 77.8, 50.0 and 80.0%, respectively, in the three ACA groups. Based on the probability of correct grouping, the AUC of the model efficiency determined by ROC analysis was 0.740. Weighted kappa value was 0.772 in comparing actual and predicted groups. Quadrants were regrouped into ACA open (open angle, grade I and II) and closed (grades III and IV) groups based on gonioscopic findings. After modeling, using the binary logistic regression, the precision of the ACA open-closure model was 82.9% using LID value.

**Table 3:** Comparisons and summaries of the test data (*n* = 58)

Variables	SA	MA	LA	*P* Value
Age, years	56.1 ± 10.8	58.6 ± 7.6	56.9 ± 8.5	0.706
Male gender, %	55.6	21.4	50.0	0.121
Quadrant No. (%)	18	14	26	
Superior	6 (33.3)	3 (21.4)	5 (19.2)	
Inferior	4 (22.2)	5 (35.8)	6 (23.1)	
Nasal	3 (16.7)	3 (21.4)	9 (34.6)	
Temporal	5 (27.8)	3 (21.4)	6 (23.1)	

*SA* = small angle; *MA* = moderate angle; *LA* = large angle

**Table 4:** Prediction of correct number and percentage of quadrants based on the multiple ACA grades model

		Predicted/Actual, No./No. (%)		
		SA	MA	LA
Actual Groups	SA	14/18 (77.8)	3/18 (16.7)	1/18 (5.5)
	MA	1/14 (7.1)	7/14 (50.0)	6/14 (42.9)
	LA	0/26 (0.0)	5/26 (19.2)	21/26 (80.8)

*SA* = small angle; *MA* = moderate angle; *LA* = large angle

**Fig. 5:**
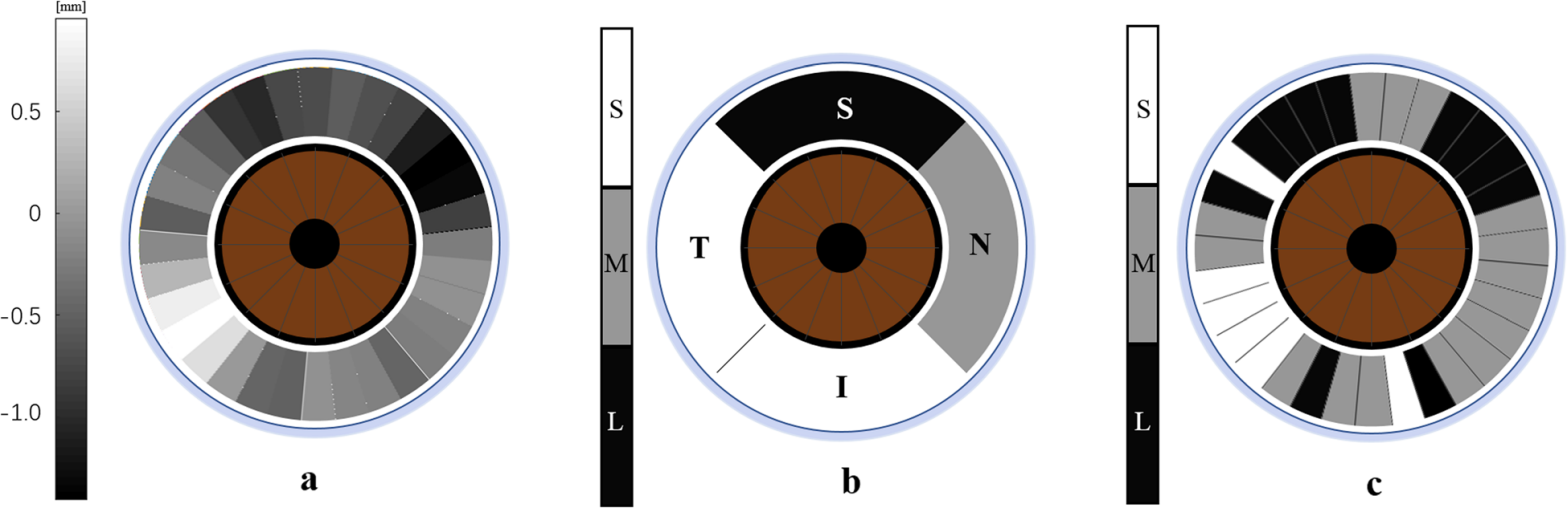
Angle distribution of the right eye of a 60-year-old male. **a** LID map. **b** Grading results of four quadrants based on the model. **c** Grading map of every 11.25° of ACA based on the model. The bar in *a* represents the range of LIDs. The bars in *b* and *c* represent the three groups of ACAs, i.e., black, small angle; gray, moderate angle; white, large angle. S, superior quadrant; I, inferior quadrant; T, temporal quadrant; N, nasal quadrant

By radial scanning, the distribution of LIDs could be mapped for each scan set (Fig. 5a). After averaging and grouping, the grading results of the four quadrants, which were similar to the gonioscopy results (Fig. 5b), can be obtained for the purpose of clinical diagnosis. The ACA in the superior quadrant of this eye had a small angle, while the nasal quadrant had a moderate angle, and the inferior and temporal quadrants had large angles. Furthermore, using the multiple ACA grades model, each 11.25° interval of the ACA can be assigned into one of the three groups, with higher spatial resolution than gonioscopic findings (Fig. 5c).

## Discussion

We used AS-OCT and image processing programs to obtain images of the anterior chamber structure and determine accurate measurement of the parameters associated with it. For patients, the examination was quick and convenient, and other studies have shown that AS-OCT with image processing programs have lower error rates due to the reduction of human errors during the examination of the gonioscopy [30]. Meanwhile, AS-OCT images and the computations derived from them provide sufficient information to determine if the ACA is closed. In our study, both AOD750 and TISA750 had high AUC, which was in line with a previous study [26]. However, AOD750 and TISA750 were excluded to establish the model through a step by step regression. The new parameter LID had a high AUC in two-two comparison and was applied to establish the multiple ACA grades model. Variations of iris contour were important for the assessment of the grading model. Not only the region of 750 μm anterior to the scleral spur should be taken into the consideration, but also the whole iris configuration was important for angle grading. LID, which was defined on the basis of gonioscopic optical path, reflected the whole iris configuration with the tangency to the vertex of the iris. To the best of our knowledge, with both high intraobserver and interobserver agreement (Fig. 3), the new parameter that we have designated as LID has not been previously reported.

Compared with SA and LA, the correct rates of MA (50%) was relatively low (Table 4), however, it was still higher than the guess rate of 33.3% for three groups. This could be explained by the difficulty in observation of the adjacent gonioscopic anterior chamber structure, on which the grouping criteria was based. The precision could be increased when quadrants were divided to groups of open angle and angle closure compared with the three-group model. Agreement was also fair to moderate in multi-grade scheme however slightly higher with the open-closed scheme in the work of Phu et al. [31], who compared the ACA structure using gonioscopy and AS-OCT. Even so, the advantage of the additional MA group demonstrated in this study could not be underestimated. Yip et al. found that there was a difference between patients with incident PACS and no PACS in the cumulative Shaffer grade (the sum of all Shaffer grades identified in each of the four quadrants) [32]. Those with a cumulative Shaffer ≤8 had a relative risk of 4.55 for developing PACS, combined with the grouping model in this study, indicating a relatively high risk of angle closure for MA, compared with angles in the group of LA. Therefore, the multiple grading method could provide guidance for follow-up time by future longitudinal studies in clinic.

Once the ACA grades model was built, ACAs at each imaging position by AS-OCT can be evaluated, leading to the ACA mapping with higher spatial resolution compared with gonioscopy. As shown in Fig. 5c, model findings of multiple grades at 32 position of 360° circumferentially can be provided with this scanning mode, while the gonioscopy only reveals grading results of four quadrants. Furthermore, the proportion of small chamber angles in the whole eye can be measured based on the detailed grading map. For instance, the small angles in Fig. 5c occupied 34.4% of the total ACA. The high-speed AS-OCT (200 kHz) employed in this study provides feasibility for high density radial scanning without artifacts in OCT images caused by eye motion.

There are some limitations in this study. Although this study showed that AS-OCT could clearly image the entire chamber angle and precisely locate the scleral spur, this precision might be lower during clinical examinations, especially if the operator does not have sufficient experience in identifying the scleral spur [33]. At present, manual input of the scleral spur location is required due to the semi-automatic image processing program, which can add to the error rate. However, this situation can be improved upon the increased use of big data machine learning with auto-recognition of the scleral spur. Besides, the conventional gonioscopic criteria was applied with the assessment of the narrow quadrants of the whole eye, whereby the superior quadrants were commonly quite narrow [34, 35]. However, vertical quadrants could not be completely imaged in every single eye in this study as the eyelids were difficult to pull apart in those with small palpebral fissure, and hence, circumferential evaluation of the ACA could not be applied for a few individuals using this OCT grading model. Another shortcoming of the study is that the multiple ACA models can only represent the static classification of the gonioscopy but cannot show dynamic classification results. In future studies, indentation examination could be applied by AS-OCT with the variation of ambient brightness.

## Conclusions

This study demonstrated that, compared with gonioscopy, AS-OCT provided a non-contact, fast, high resolution, and quantifiable examination for ACA inspection. With a low measurement variability, the newly-defined parameter LID could be feasible to establish the first AS-OCT-based multiple ACA grades model for quantitatively evaluating the ACA and providing a methodological basis for subsequent studies. This classification model could be applied in both angle closure screening and clinical application longitudinally, and could potentially add clinical and commercial value.

## Data Availability

All data generated or analyzed during this study are included in this published article.

## Abbreviations

PACG: Primary angle closure glaucoma
IOP: Intraocular pressure
AS-OCT: Anterior segment optical coherence tomography
ACA: Anterior chamber angle
LV: Lens vault
AOD: Angle opening distance
TISA: Trabecular-iris space area
SA: Small angle
MA: Moderate angle
LA: Large angle
LID: Light intersection distance
AUC: Area under the curve
ROC: Receiver operating characteristic
